# Holding and rupture: Describing post-traumatic stress among former UK Army and Royal Marine personnel deployed to Iraq and Afghanistan

**DOI:** 10.1371/journal.pone.0308101

**Published:** 2024-08-09

**Authors:** Laura Palmer, Walter Busuttil, Amos Simms, Nicola T. Fear, Sharon A. M. Stevelink

**Affiliations:** 1 King’s Centre for Military Health Research, Department of Psychological Medicine, Institute of Psychology, Psychiatry and Neuroscience, King’s College London, London, United Kingdom; 2 Academic Department of Military Mental Health, King’s College London, London, United Kingdom; 3 Department of Psychological Medicine, Institute of Psychology, Psychiatry and Neuroscience, King’s College London, London, United Kingdom; The Quinism Foundation, UNITED STATES OF AMERICA

## Abstract

Former UK military personnel who were previously deployed to Iraq and Afghanistan in combat roles have exhibited elevated levels of Post-Traumatic Stress Disorder (PTSD) compared to other groups. The present qualitative analyses used semi-structured interviews and a framework analysis to compare the experiences of symptomatic (N=10) and asymptomatic (N=7) former Army and Royal Marine personnel who were exposed to combat. Participants were drawn from a large UK military health and wellbeing cohort study and were sampled based upon probable PTSD status using scores from the PTSD Checklist-Civilian Version (PCL-C). All symptomatic participants attributed the development of post-traumatic stress to deployment events, with one additionally ascribing symptoms to childhood events. Among the participants, post-traumatic stress was temporarily buffered, and held at bay, by the *holding* function of various military structures, including the military collective; cultural and ethical frameworks that helped to organise traumatic experiences; an operational necessity for psychological compartmentalisation and even the distraction of deployment itself. Leaving the military appeared to elicit a global *rupture* of these supports. As a result, the military-to-civilian transition led to an intensification of post-traumatic stress, including deployment-related memories, among the symptomatic participants. In contrast, asymptomatic participants tended to report continuity of their *holding* structures across the lifespan, especially across the military-to-civilian transition. The onset and maintenance of post-traumatic stress may thus be explained by an interplay between the capacity of *holding* structures and the magnitude of lifetime *rupture*. Overall, findings might provide an explanation for the widening discrepancies between those with enduring post-traumatic stress and those without and further research is required to determine the fit of our findings for other groups and contexts. This approach further illustrates the need to situate individual experiences of post-traumatic stress in wider structural, ecological, cultural and ethical contexts.

## Introduction

Throughout their careers, military personnel experience a series of exceptional challenges, which can include the culture-shock of enlistment, combat deployments and the complex practical and relational challenges of leaving the military [1–9]. Over the past two decades, research efforts have been dedicated to assessing the impacts of these extraordinary life experiences upon military personnel. It is consistently found that most military personnel who experience stressful and potentially traumatic events do not develop ongoing mental health problems [9–13], however a substantial minority report serious and enduring psychological and social difficulties, particularly once having left the military [14]. A cohort study of current and former UK military personnel found that levels of probable PTSD were comparable to the general population in England (4-5%) in its first two phases (2004-6 and 2007-9) [15, 16], but increased to 6% overall in 2014-16 [9]. It appeared that levels were particularly elevated in some subgroups, including regulars who were previously deployed in combat roles to Iraq or Afghanistan and had since left the military (17%) [9]. Whilst levels of probable PTSD are elevated in this group, the majority do not meet thresholds for probable PTSD, beckoning the question of why individuals who are exposed to similar events might be differentially impacted.

The diagnostic definition of PTSD outlined in the Diagnostic and Statistical Manual of Mental Disorders-V hinges upon a central external causal event (Criterion A: Exposure to actual or threatened death, serious injury, or sexual violence) [17]. It is, however, a coalescence of various biological, cognitive, social, and intrapsychic factors that determine whether an initial stress response crystallises into a persistent disorder [18, 19]. During the traumatic event, an individual’s stress-response systems may be overwhelmed by a multisensory activation, creating a state of reactive dissociation [20]. The normal encoding of memories is then disrupted, meaning traumatic memories are not properly integrated, are fragmented and biased toward the emotional and sensory qualities of an event [21–24]. However, the development of PTSD can also be influenced by factors that predate and extend beyond the event itself. For instance, childhood adversities are predisposing factors by modifying the development of an individual’s general stress reactivity, emotional regulation, coping styles and cognitive beliefs [25, 26]. Post-event, the development of a disorder might be further informed by how traumatic memories are modulated and (re)consolidated [22, 27], the meanings given to such events [28, 29], and the degree to which an individual can access social support [21].

Research into the aetiological mechanisms of PTSD might be further enhanced by adopting a phenomenological posture to explore the lived experience of symptoms. In military contexts, a growing body of work has sought to qualitatively investigate a range of phenomena, from moral injury [30, 31], post-traumatic growth [32], military sexual trauma [33, 34] to the experience of living with a partner with PTSD [35–37]. By committing to a study of the lived experience of a phenomenon, and the feelings, sensations and interpretations that arise, such studies can give insight into ‘on-the-ground’ details of their participants’ psychological processes. Other work has more explicitly sought to delineate the phenomenology of certain disorders by reflecting upon what is like to subjectively experience symptoms and the kind of ‘world-experiences’ these generate [36, 37]. For example, the ‘depression-experience’ may involve alterations in time-consciousness, (i.e. the rate at which time passes or feeling a detachment from time), changes in one’s existential feelings and sense of possibility, and an essential estrangement in interpersonal relationships [38]. In a similar vein, the eruption of strong, active memories of the past during a PTSD flashback can disrupt individuals’ experience of a linear flow of time [39]. Such a lens can expand an understanding of a disorder by allowing for the identification of distinct and elemental features. Whilst understanding the lived realities of disorders is the mainstay of clinical practice, Wilde (2022) notes that *“little research… focuses explicitly on the details of how a potentially traumatizing event is experienced and how this experience relates to the development of subsequent distress”* (p. 690) [40].

The life-world of the individual includes their wider social and political circumstances. In the UK military context, the development of post-traumatic stress may occur within and across a range of different environments, from military training, military culture, deployments and the military-to-civilian transition. For the cohort focused upon in this study, post-traumatic stress reactions were linked to the specific contexts of the Iraq and Afghanistan conflicts. These conflicts were initiated by the United States (US) as time-limited operations in the name of exogeneous state-building, yet they continued for twenty years and brought a host of challenges, including counter-insurgency warfare, the unprecedented use of Improvised Explosive Devices (IEDs) and many casualties and fatalities of military personnel and Afghan civilians [41–44]. Since this study was conducted, the Taliban has seized Afghanistan and formed an interim government, re-establishing much of its repressive legislature and worsening the humanitarian crisis in Afghanistan. Whilst the repercussions of the Afghanistan conflicts are still unfolding, renewed media attention and concerns about the ethics and justifications of the conflict may elicit, or intensify, distress among those involved in these deployments [45]. Post-traumatic stress, its meaning and how its experienced, is thus inextricable from evolving political and cultural contexts.

This study thus sought to explore the development of post-traumatic stress in a specific subgroup with the highest levels of probable PTSD in the UK Armed Forces community [9]. These included former UK military personnel who had deployed in combat roles to Iraq or Afghanistan during their time in the military. The study’s objectives were 1) to explore how traumatic stress emerged over pre-enlistment, peri-military and post-military periods, and 2) to compare the life experiences and psychological reactions of those with probable PTSD symptoms to those without.

## Materials and methods

### Sample

Participants were drawn from the King’s Centre for Military Health Research’s longitudinal study, which was initiated in 2004 to examine the health and wellbeing of UK Armed Forces personnel after the start of the conflict in Iraq (2003). Data collection for phase 1 occurred in 2004-6 (N=10,222 total respondents) [15]; for phase 2 in 2007-9 (N=9,990) [16] and for phase 3 in 2014-16 (N=8,093) [9]. Participants were eligible to take part in the current qualitative study if they were ex-members of the regular force who had deployed to the Iraq or Afghanistan conflicts in a combat role and had participated in phase 3 of the cohort study. This was so that sample reflected the characteristics of the subgroup with the highest levels of PTSD found in phase 3 [9].

In total, we interviewed 17 participants (10 in the symptomatic PTSD sample, 7 in the asymptomatic PTSD sample). Participants were assigned to the symptomatic sample if they scored ≥ 50 on the PTSD Checklist-Civilians (PCL-C) [46] according to their phase 3 questionnaire responses. Participants were asymptomatic if they reported no, or minimal, symptoms at all available time points. Caseness on the PCL-C was used as a grouping principle to represent the presence and absence of traumatic exposure related symptoms. In order to ensure that asymptomatic cases had not become symptomatic since phase 3, we re-administered the PCL-C before the interview took place. Whilst the symptomatic/asymptomatic status of the sample was defined by caseness for probable PTSD, the interviews captured a breadth of intrapsychic and reflective processes beyond the specific sequalae of PTSD. This was inevitable since PTSD is highly comorbid with other mental health issues [47] and as symptoms were embedded in intricate narratives about an individual’s life experiences and inner lives.

Participants were eligible to take part in this study if they had consented to learn about future research during their participation of the cohort study and if they lived in the UK. The latter criteria enabled us to follow the study’s risk protocols responsively and responsibly (see section on ‘Procedure’). The sample was restricted to Army or Royal Marine personnel in order to obtain a more homogenous sample with more typical ‘front-line’ combat experiences. We aimed to recruit women in the sample in order to include female perspectives and were able to interview two women (one in each sample). The two samples were matched approximately on their levels of combat exposure; this was determined from a list of combat experiences endorsed in their cohort questionnaires. This ensured that the differences between the sample were not simply due to experiencing fewer potentially traumatic events in combat. Sample characteristics are outlined in the Supplementary materials (S2 File).

### Procedure

Data collection began in September 2019 but was prematurely terminated due to the COVID-19 pandemic in March 2020. This was led by concerns that participants might not have had the emotional resources to take part in interviews about traumatic experiences or have had access to confidential spaces or support services during the first more restrictive lockdown phase. On review of the data we had collected, failure to reach our target sample size (N=15 in each group) was offset by the richness and detail provided by trauma histories already collected. The larger size of the symptomatic sample was acceptable since these participants exhibited greater diversity in experiences and a larger volume of data by virtue of experiencing symptoms.

Eligible participants were emailed or posted a study invitation pack including a Participant Information Sheet, a Consent Form and a booklet of relevant signposting services. The main researcher (LP) contacted interested participants to determine their eligibility and arranged telephone interviews at the participants’ convenience. Before the interview, written consent was obtained, and participants completed an online survey repeating the PCL-C and asking about historical/current mental health conditions. This verified the asymptomatic status of the comparator group and provided relevant information for the interviewer (LP) to reference during interview.

Interviews ranged from 1 to 2.5 hours. A semi-structured interview guide provided an adequate structure across three periods: pre-, peri- and post-military life, whilst also giving participants the freedom to bring up other parts of their biography when this was meaningful [48]. The interview started with participants’ military careers, experiences of transition, post-military life and ending with their childhood experiences (see S3 File for the general interview guide followed). This order allowed for a rapport to develop between the main researcher (LP) and participants before discussing sensitive events which, within this sample, included childhood sexual abuse.

To ensure the safety of all participants, a robust risk protocol was followed. During the interviews, the interviewer was attentive to how participants’ responded to the interview questions, including listening out for changes in tone, hesitations and signs of being upset, and provided reassurance when needed that participants could move on to another question, pause or stop the interview at any point and could withdraw from the study up until their data had been analysed. Participants were offered a clinical call-back if they exhibited distress. If participants indicated an immediate risk to themselves or others, the researchers would contact relevant authorities, although this was not necessary in this study. The main researcher was also able to access clinical support and supervision after interviews and during analyses in order to debrief and reflect upon their experiences. Ethical approval was obtained via the King’s Psychiatry, Nursing and Midwifery Research Ethics Subcommittee (Ref: HR-18/19-11668).

Interviews were audio-recorded and transcribed by an independent transcription company that had signed a confidentiality agreement. Participants’ audio data and transcripts were stored under a unique identifier and separate to identifying details. Audio data were destroyed at the end of analysis and transcripts were pseudonymised. To ensure their anonymity, pseudonyms were used in the write-up of results.

### Analysis

The focus of the analysis was to track the emergence of distress or post-traumatic stress as it occurred in interaction with events and dynamics across the lifespan. A framework approach, as outlined by Ritchie and Spencer (1995), provided practical steps for analysing and identifying patterns across a large volume of data across multiple cases [49].

The first step was to develop an initial framework directing the interview guide and acting as an overarching structure for raw data. The framework had a chronological structure spanning pre-enlistment, military and post-military periods, and thematic categories included stressors, psychological responses and contextual information within each period. A second step involved the main researcher (LP) familiarising with audio and transcript data; a third step was the development of codes from the data using Nvivo, v. 12 software, and a fourth step involved grouping codes into themes.

At an early stage of analysis, it was apparent that the framework was not capturing the crux of the symptomatic and asymptomatic samples’ differences. Whilst the samples reported similar life events, they were evidently discrepant in their psychological responses and outcomes. The framework was then revised to better track interrelationships between events, psychological responses and wider life circumstances by grouping codes into various ecological levels (‘individual’, ‘relational’ and ‘institutional/societal’) and developing bespoke visual timelines for each individual. The latter technique performed the fifth step of charting, which ordinarily involves building a separate database that summarises all cases. Visual timelines allowed for a more agile assessment of lifetime patterns within, and between, the symptomatic and asymptomatic samples. Biographies were aligned to allow for the cross-case inspection across key events, e.g., the intensification of the Afghanistan operations. The coding of data was undertaken by the main researcher (LP); insights arising during data analysis and the formation of themes were discussed iteratively between the study authors. A reflexivity statement outlining a summary of the researchers’ experiences is included as Supporting Information (S1 File).

Throughout the analysis, particular attention was given to how participants described their distress, thus chiming with a phenomenological focus upon individuals’ subjective experiences of ‘symptoms’. Therefore, whilst a phenomenological methodology was not formally employed in the present study, the analysis was informed by an aspiration “*to capture as closely as possible the way in which the phenomenon is experienced within the context in which the experience takes place*” (p. 27) [50]. From this perspective, post-traumatic stress needed to be understood in relation to individuals’ lives as a whole [51].

## Results

### Summary

A table of characteristics can be found in the Supporting Information (S2 File). The average age of the symptomatic and asymptomatic samples was 41 years (ranging from 31 to 51 years). In total, 15 participants were men and two were women (n=1 symptomatic and n=1 asymptomatic sample).

Seven of the 10 symptomatic participants, and five of the seven asymptomatic participants had enlisted before the age of 18 years. We interviewed three commissioned officers in the asymptomatic sample, however no officers were included in the symptomatic sample. This imbalance was due to the premature termination of data collection due to COVID-19. Both samples had deployed to combat or peacekeeping missions four times on average (excluding training exercises). All participants had deployed to the recent Iraq or Afghanistan operations. Six out of 10 of the symptomatic participants and three out of seven of the asymptomatic participants had deployed to the First Gulf War, Northern Ireland, Bosnia or Kosovo. The samples spent a similar length of time in the military; on average, 15 years (range 4-26 years) in the symptomatic sample and 12.7 years (5-23 years) in the asymptomatic sample. Four out of 10 of the symptomatic participants and one out of the seven asymptomatic participants had left the military via medical discharge for physical injuries and one symptomatic participant reported a comorbid psychiatric diagnosis.

Participants were classified as symptomatic/asymptomatic based on their PCL-C scores (≥50) from their cohort questionnaire data. We administered a pre-interview survey asking participants about any known mental health conditions. Nine of the 10 symptomatic participants reported having PTSD in their survey or interview; the remaining participant indicated a diagnosis of depression. None of the asymptomatic participants were cases on the PCL-C or indicated a diagnosis of PTSD pre-interview.

All symptomatic participants attributed current mental health problems to deployment events that met the threshold for criterion A, i.e. involving the exposure to actual or threatened death, serious injury, or sexual violence. Three participants could not isolate specific events; for example: “*I can’t put it down to one thing*. *It’s an accumulation of everything*” (Chris, symptomatic sample). Six of the 10 symptomatic participants continued to deploy after experiencing their criterion A event(s).

Nine of the 10 symptomatic sample reported childhood adversities ranging from strained family relationships to physical and sexual abuse perpetrated by fathers. Other stressors included growing up in a violent community and homelessness. Only one participant attributed their PTSD to events from both childhood and deployment. Three of the seven asymptomatic sample reported childhood adversities including sexual abuse.

The Results section will now present the themes and subthemes relating to each research objective (as summarised in Table 1).

**Table 1:** Themes and subthemes in relation to the research objectives.

Research objective	Themes	Subthemes
**Research objective** 1: To explore how traumatic stress emerged over pre-enlistment, peri-military and post-military periods.	Compartmentalisation	
	Episodic collapse	
	Conscious realisation	
**Research objective 2:** To compare the life experiences and psychological reactions of those with probable PTSD symptoms to those without.	The military *holding* environment	• A (familial) collective • Ethical and cultural sense-making systems
	The paradox of deployment	• Deployment ruptures: i) Visceral and vivid exposures ii) Ethical dissonance iii) Prevention of real-time processing iv) Revealing the limits of training • “*I was in my element*”
	Leaving the military: Global *rupture*	• The decontextualisation of trauma • Holding begets holding; rupture begets rupture

### Lived experiences of post-traumatic stress

In the following section, we outline the findings relating to research objective 1 regarding participants’ lived experiences of the development of post-traumatic stress. Three themes were generated to reflect this process: ‘*Compartmentalisation’*, *‘Episodic collapse’* and *‘Conscious realisation’*.

#### Compartmentalisation

The first theme, ‘compartmentalisation’, was evident in the narratives of both samples. Compartmentalisation referred to the automation of military training in deployment settings and the subsequent bracketing, or suppression, of immediate emotional responses in the face of stressful events. This was considered an operational skill by many participants and was deemed an essential part of the military toolkit:

*“You have this professionalism. You just blank out all emotion type of thing but then when you get back it’s trying to let it go if you know what I mean”* (Chris, symptomatic sample)

“*At first*, *I completely shut off to [the stress] because… the training that you had done*, *the repetitive nature of it*, *just kicks in straight away and I just cracked on with what I had to do”* (Freddie, symptomatic sample)

*“You just had to crack on and get on with the job”* (Craig, asymptomatic sample)

‘Compartmentalisation’ was regarded by some asymptomatic participants as the primary reason for remaining psychologically unaffected by events on deployment: *“I had a shelf*, *if that makes sense*. *Once I left [deployment]*, *I just put that to one side”* (Gareth, asymptomatic sample). In contrast, it was common for symptomatic participants to view ‘compartmentalisation’ as a pre-condition, or their ‘baseline’ state before the eventual development of post-traumatic stress. In this way, symptomatic participants described how they “*boxed*” (Dan, symptomatic sample), “*bottled*” (Tom, symptomatic sample) or “*masked*” (Mike, symptomatic sample) the impact of traumatic events or had become “*emotionally closed off*” (Matthew, symptomatic sample). Most symptomatic participants did not register changes in their psychological state or behaviours after their initial deployments, even if this was when their criterion A event(s) had occurred. For example, when asked about his criterion A event, Jimmy (symptomatic sample) stated that the impacts were not felt “*immediately it was just*, *like I say it was a couple of years down the line”*.

Some viewed the ability to compartmentalise as a function of young age and naivety: “*I was young enough I kind of absorbed everything and took on board this was normal*” (Steve, asymptomatic participant); yet, contrary to this, ‘compartmentalisation’ was also considered as the result of experience and having habituated to conflict exposures: “*it sounds horrendous*, *but you kind of got used to seeing all this”* (Keith, asymptomatic participant). Overall, compartmentalisation allowed some participants to continue deploying with residual, or unresolved, post-traumatic stress.

#### Episodic collapse

‘Episodic collapse’ was a second stage for some asymptomatic and most symptomatic participants. ‘Episodic collapse’ related to the momentary collapse of ‘compartmentalisation’, manifesting as angry outbursts, e.g. “*going from zero to ten*” (Freddie, symptomatic participant); personality changes (“*Jekyll and Hyde*”: Chris, symptomatic participant); acting “*out of character”* (Steve, asymptomatic participant); a lack of care for consequences; nihilistic and fatalistic thinking that led to lack of care for oneself, others and reckless behaviour including drink-driving, seeking sex and fighting: “*It was guaranteed I’d be in a fight because I just wasn’t bothered and I wasn’t bothered if I hurt him*, *if you know what I mean*?” (Tom, symptomatic participant). Participants also reported nightmares and emotional numbing, which was aided by alcohol: *“Alcohol numbs all of my feelings*. *I didn’t really feel at all”* (Beth, symptomatic participant).

As already noted, these initial signs of post-traumatic stress rarely occurred after participants’ initial deployments. ‘Episodic collapse’ was reported by both samples. These incidents seemed to be more common and serious among the symptomatic participants, and included suicide attempts and violent threats:

*“I saw a little bit of the red mist and actually pulled my pistol on X… And I suddenly realised that I wasn’t 100%”* (Chris, symptomatic participant)

*“I took an overdose a few weeks after I got out and ended up in hospital for a week*. *Again*, *they asked me there… but it’s just hard to pinpoint what was actually the problem*. *I knew I wasn’t myself and I knew there was something going on with me”* (Ali, symptomatic participant)

At this stage, participants did not always connect mood instability and changes to their personality with experiencing post-traumatic stress:

“*I didn’t realise anything about mental health. I didn’t realise why things are happening… I just got through it, which then after… maybe 3½ years later, everything came to a basically downward spiral where it was uncontrollable*” (Jimmy, symptomatic participant)

Collapse, for some, was sudden, i.e. “*I don’t think there were any warning signs” (*Brandon, symptomatic participant) and tended to abate over time. This was most common among asymptomatic participants who reported an initial stress reaction. For others, collapse referred to a longer-term erosion of ‘compartmentalisation’: it “*took a little bit of time*. *It crept up”* (Beth, symptomatic participant), *“[it was] lingering”* (Freddie, symptomatic participant) in the background, or, as Ali (symptomatic participant) states: “*I think there was always something going on*. *Never settled in myself*. *Never that happy to just settle and be relaxed*.*”*

Commonly, instances of ‘episodic collapse’ temporarily resolved or remained manageable and participants continued to deploy.

#### Conscious realisation

With time, ‘compartmentalisation’ began to slowly rupture and leaving the military marked its virtual entropy. This gave rise to a third state which we have termed ‘conscious realisation’. ‘Conscious realisation’ referred to the individual joining-up their current distress with past deployment experiences. This was characterised by noticing more intrusive traumatic memories, nightmares and a persistently thinking through specific events:

*“Nothing has really sunk in until the past few years. It just seems to be years after that things I’ve realised, or I’ve thought things*” (Jimmy, symptomatic sample).

*“Considering I joined up in [date]*, *it [event featured in flashbacks] didn’t come to the surface until [over twenty years later]*. *And that’s when it all started coming to a head… it was only later… I had flashbacks a couple of years ago after I left service*. *I’d just zone out and sometimes in the weirdest places*. *Sometimes the trigger would just be a smell*, *even now I can’t go down the raw meat aisle of Tesco*” (Matthew, symptomatic sample).

Among some participants, ‘conscious realisation’ manifested as physically re-experiencing or re-enacting combat events:

*“I wake up in the middle of the night wriggling all over the place… I can taste the area, I can smell the area, I can even go through the motions of trying to do a tracheotomy (making an incision in the neck to insert a tube to support breathing). So that one affects me” (*Mike, symptomatic sample).

#### Comparison of the samples

Some asymptomatic participants described ongoing protective defences that continued to shield them from the impacts of their deployment experiences. These included Gareth’s (asymptomatic participant) aforementioned “*shelf*” and Craig’s (asymptomatic participant) disposition for being unimpacted by stressful events: “*I didn’t feel I needed to do any coping mechanisms*, *do you know what I mean*? *I’ve always been… very robotic in some ways*”.

Other participants described processing deployment events as they were happening: “*I was definitely processing while I was there*.” (Gareth, asymptomatic participant) and “*I have a very calm quiet response*. *I think that’s just me when things like that happen*. *But I don’t bottle them up*. *I remember absolutely bawling my eyes out*” (Rachel, asymptomatic participant). Others reported different degrees of ‘working through’ their deployment experiences. Jordan (asymptomatic participant) recalled journaling and reading war literature and memoir. Steve (asymptomatic participant) described debriefing with his wife after every deployment: “*I’ve used that method ever since on deployment*. *I’ve always sat down after the tours and sat down with my wife*”.

Asymptomatic participants appeared to more easily access support during or after their deployment(s). This included speaking with their friends, colleagues, family members and/or interventions like Trauma Risk Management (TriM). In contrast, some participants in the symptomatic sample explained how emotional numbing and disorientation prevented them from recognising or articulating something was wrong. As Brandon (symptomatic participant) describes: “*I just couldn’t talk”*. Ali (symptomatic participant) similarly remembers:

*“It was hard to explain because people did ask but I could never explain it… it’s just hard to pinpoint what was actually the problem”.*

### Holding and rupture

So far, we have explored how post-traumatic stress developed according to the lived experience of the participants we interviewed. The second research objective sought to explain the differences in responses between the asymptomatic and symptomatic samples.

Overall, our analysis indicated that no singular exposure or remedial support was responsible for the samples’ differences. Instead, a deeper analysis led to the development of two concepts from the data, which we have termed *holding* and *rupture* to help explain diverging patterns among the samples’ experiences. *Ruptures* referred to stressful or traumatic events, transitions and dynamics which created disturbances for individuals or required adaptation. *Holding* referred to the function of supports in buffering and helping to contain *ruptures* over time. From reviewing patterns within the sample’s interview data, it seemed the emergence of post-traumatic stress thus hinged upon a through-life interplay of *holding* and *rupture*.

Examples of this process are described by three main themes, namely: ‘The military holding environment’, ‘The paradox of deployment’ and ‘Leaving the military: Global rupture’.

#### The military *holding* environment

In summary, the military seemed to provide a *holding* environment for both samples to quite successfully ‘compartmentalise’ the effects of difficult life experiences. The *holding* capacity of the military can be broken down into multiple scaffolds. These holding structures appear to span institutional, cultural and individual levels. These are summarised by the following subthemes: ‘a (familial) collective’ and ‘ethical and cultural sense-making systems’. Over time, the *holding* capacities of the military began to wane and no longer supported the *ruptures* experienced by individuals. This escalated in the years after leaving the military where most supports appeared to *rupture*. Alternatively, most asymptomatic participants appeared to sustain or replace such supports, especially across the military-to-civilian transition. The following sections explains how *holding* and *rupture* played out over deployment and post-military settings.

*A (familial) collective*. The subtheme, ‘a (familial) collective’, references how the military fulfilled participants’ basic needs (i.e. education, healthcare, food and shelter), in addition to offering kin-like relationships with leadership and peers, which some participants described using quasi-familial terms:

“*I was brilliantly looked after by very capable non-commissioned officers… I was taken under the wing of a very competent platoon sergeant”* (Jordan, asymptomatic participant).

*“You become part of the brotherhood and it was fantastic”* (Keith, asymptomatic participant*)*.

The military collective was further described by Keith (asymptomatic participant) as a “*safety net”;* the effectiveness of which was explained by Jordan (asymptomatic sample) when he was separated from his unit after returning from an Iraq deployment:

*“I felt removed from my support group, people I’d trained with, people I’d been living with for months and fighting alongside and those very, very tight emotional forged relationships that you develop under fire I was suddenly cut out from them and extracted from them and isolated from them”.*

This environment was particularly supportive for participants with family difficulties like Matthew (symptomatic participant) who had experienced childhood physical abuse and homelessness before enlisting. The military’s wrap-around supports appeared to *hold* and harness Matthew’s desire to seek risk, and tolerance of stress and violence stemming from his childhood. He explained how childhood physical abuse *“gave me a reasonably decent survival instinct*, *how to take a beating and keep on ticking… And it served me*. *Yes*, *very early*. *And I would say it stood me in good stead… the only thing is now I can’t switch off”*. In this regard, the early tendency toward withstanding adversity by ‘switching off’ appears somewhat congruent with the requirement to ‘compartmentalise’ (as described on p. 12) and enabled him to deploy to many conflict zones throughout the 1990s-2010s.

*Ethical and cultural sense-making systems*. The final subtheme relates to the military’s ‘ethical and cultural sense-making systems’. These systems provided ways of thinking, norms and ideologies that helped participants to psychologically manage and frame their individual role on deployments and the *ruptures* they encountered. Part of this is noted within the theme ‘compartmentalisation’ (p. 12) where participants described professional capacities, or a toolkit, which provided a system to handle the intensity of stimuli in deployment settings. For example, Ali (symptomatic) described how the automation of training was protective in helping him to manage the magnitude of responsibility he had for the welfare of other personnel:

*“At the end of the day it’s your decisions that is going to impact the outcome. Your mistake could cost your men their lives. So it is very, the responsibility is very big but that’s where you rely on your training and everything to kick in”.*

Sense-making systems further extended to ideological framings of an operation. Believing in what one was doing on deployment therefore had a *holding* potential:

*“[On being in Afghanistan] We made a positive impact and I think if I didn’t feel that, it would sit with me very, very differently today having… seen some of the things I’ve seen”* (Rachel, asymptomatic participant).

“*[When asked about how the participant felt post-deployment]*: *Fine because we felt like we’d achieved something*. *So it was nice coming home and we felt like we achieved something on the tour*. *Second one not so much”* (Dan, symptomatic participant).

Conversely, when sense-making systems did not successfully justify violence and loss, participants struggled to process traumatic events. For example, Mike (symptomatic participant) reflected upon his criterion A event:

*“I don’t know why that one affected me. I’d even seen worse than that… I don’t know whether it’s because he was reasonably young… Is it because when we came back and we’d not found any weapons of mass destruction? I thought, well, that was a needless death”.*

Operational language was another resource that supported ‘ethical and cultural sense-making’. Despite having left the military, deployment events were often recalled by asymptomatic participants using operational terminologies, less graphic descriptions and had a less overt emotional quality:

“*We did a few strike operations as well. Intercepting high priority targets… Again, it was on and off kinetic*” (Keith, asymptomatic participant).

There was also a tendency among asymptomatic participants to refer to the group’s reaction (and using ‘we’ instead of ‘I’) when asked about personal impacts. For instance, when asked about the personal impact of a specific deployment event, Craig (asymptomatic participant) shared: “*that was hard for all of my troop”*. Shocking events were further described by asymptomatic participants using descriptors of ‘a bit’ or ‘quite’, ‘bizarre’, ‘unusual’ and ‘weird’.

Another facet of the military’s ‘ethical and cultural sense-making systems’ was the need to make in situ assessments about the value of life. Dehumanisation, a timeless strategy in conflict, appeared to support ‘compartmentalisation’ on deployment. Keith (asymptomatic participant) explained:

*“When you are in the middle of a combat situation you are looking at the most effective way of destroying the enemy and cutting them down. You become extremely clinical… and you feel no compassion towards those individuals whatsoever. You just cut them down because that’s the only way you can deal with it”.*

In addition to the ‘enemy’, assessments about the value of life extended to the life of oneself and their peers. Dan (symptomatic participant) described coming to terms with his own death in favour of protecting younger colleagues:

*“The way I saw it is ‘I had a life’ so if I die I die… I was 35 at that stage, so I’ve had a life, where if you look at it the guys were 20 or 21, they were children in my eyes… If I die it’s fine I’ve had a life but you haven’t.”*

The conditions of conflict thus require an organising structure, and a kind of reasoning, which entailed the division of ‘us’ and ‘them’, self-sacrifice and a belief that one is ultimately doing something that is ‘good’.

#### The paradox of deployment

Deployments placed inordinate pressure upon the *holding* structures of the individual, their relationships and the military institution. We undertook an analysis of all criterion A events described by the symptomatic sample and the most challenging events described by the asymptomatic participants to build a picture of the incidents that caused the most *rupture*. Four characteristics were generated and these related to events on both peacekeeping and combat operations; these included events that were ‘visceral and vivid’, ‘ethically dissonant’, ‘revealed the limits of training’ and ‘prevented real-time processing’ (subthemes). This theme also houses a paradoxical dynamic. Despite being the context where most criterion A events had occurred, deployment was also an unexpected *holding* structure and was reported as supportive, positive and sometimes alleviated symptoms for some. This is explored within the subtheme “*I was in my element”*.

*Deployment ruptures*. The first characteristic, ‘visceral and vivid’, signified the highly sensory and disturbing quality of combat incidents. For example, Dan (symptomatic participants) recalled:

*“If you ever had to deal with a casualty that’s a burns victim, you can’t do anything for them… watching somebody go into shock where it changes the whole facial expression… When somebody is on fire and they inhale the flames, the noise they make you will never, ever forget”.*

The second characteristic, ‘ethical dissonance’, summarised events that strained both individuals’ and the military’s moral and ethical sense-making systems:

*“There was a f---ing 8 year old suicide bomber that came up to my checkpoint and it was ridiculous… A lot of them didn’t want to f---ing die, but they were just f----ing using them for an end…I did things that I wouldn’t necessarily agree with but it was sort of policy”* (Matthew, symptomatic participant).

The third characteristic related to events that ‘revealed the limits of training’. These were events that stretched individuals’ training and psychological toolkit beyond capacity. An example of this, and a common criterion A event, was when personnel had to provide pre-medical care to severely injured colleagues:

*“It took the back of his head off, so I was holding the back of his head… we had to bandage it up, kept him alive… he didn’t survive… That’s the main one I just keep seeing that in my head. What, if I got there faster what more could I have done? I have a lot of survival guilt. That was that one”* (Tom, symptomatic participant).

These kinds of events existed in participants’ fields as particularly ‘sticky’ or indelible; they were incidents “*that stick with you”* (Ali, symptomatic participant) and *“that will always stay with me”* (Rachel, asymptomatic participant).

The fourth characteristic referred to events that ‘prevented real-time processing’. The unyielding nature of operations meant some participants were unable to pinpoint, and therefore process, exactly what had happened. This seemed to suspend them in a continued state of confusion that they still struggled to resolve:

*“It’s difficult trying to split that tour down into just like a sequence of events… The stuff that happened was so frequent that what might happen to one person over their whole military career happened almost daily in the space of five months… So I think it’s just processing all that when you get back… I found quite difficult”* (Freddie, symptomatic sample).

*“I was in my element”*. In addition to *rupture*, deployment paradoxically enacted as a *holding* structure, particularly for those who had started encountering psychological problems. Participants in both samples described feeling rewarded and/or exhilarated by the challenge, threat and stimulus of deployment. Both samples referred to positive qualities of deployment training and of the operation in providing structure, purpose, meaning, alleviating low mood, and channelling nihilism, sensation-seeking and arousal:

*“I was so fixated, and I was so switched on in terms of my job… I got myself into a good routine, I was… in the best shape that I’ve ever been in my life… No distractions from anywhere else let’s just do my job and then I can get home*” (Jimmy, symptomatic participant)

*“I was in my element*. *I was quite happy in that situation as odd as it’s to say… I was battered and bruised all over”* (Mike, symptomatic participant)

#### Leaving the military: Global rupture

Military discharge led to profound changes for most participants. For symptomatic participants, leaving the military meant a severance from the *holding* structures containing their post-traumatic stress. Crucially, the success of the military’s *holding* capacities were already diminishing due to mounting *ruptures* from multiple deployments, physical injuries, being away from family, feelings of dissatisfaction, breakdowns in relationships with leadership and colleagues, in addition to the *ruptures* of post-traumatic stress themselves.

Being discharged from the military appeared to instigate a ‘conscious realisation’ (p. 15) of traumatic memories and experiences on deployment. This is explained by the subtheme ‘the decontextualisation of trauma’. We lastly consider the widening discrepancies between the symptomatic and asymptomatic samples through the final subtheme ‘holding begets holding, rupture begets rupture’.

*The decontextualisation of trauma*. As described, the military *holding* environment was partly defined by the ‘(familial) collective’. Ali’s (symptomatic participant) description indicates a protective function of the military collective in keeping post-traumatic stress at bay:

*“When I was back in the Army, I was OK. It was when I was away from the army that I felt [symptoms]. When I was back in camp, I was sound… I was with my mates, lads who are going through the same thing. You know what you are doing, you know what you’ve got to do. [You’re] more settled because you are where you should be… While you are in there you don’t necessarily feel it as much as when you come out and that’s when you cast off on your own”.*

The military context thus appeared to provide an environment where conflict-related trauma was mutually experienced, shared and understood. In being typical and normalised, it is possible that post-traumatic stress was hard for participants to recognise: *“[On his emerging symptoms] no one ever noticed because everyone was the same*” (Tom, symptomatic participant).

If the military collective is the proper place for deployment trauma (as Ali incisively states, “*you are where you should be”)*, then leaving the military equalled an estrangement from this sense-making context. Traumatic impact thus became personalised via the ‘individualising’ process of leaving the military (i.e. Ali’s comment “*cast off on your own”*). This may explain why participants became conscious in new ways to the memories and impacts of deployment events only once leaving the military.

*Holding begets holding; rupture begets rupture*. Some asymptomatic participants reported challenging military-to-civilian transitions, however most seemed to preserve, or recruit new, *holding* structures in their post-military lives. These structures included positive and balanced family relationships, meaningful and often military-adjacent employment, new interests, and hobbies, and old or new friendship groups. The success of supports allowed for the continuing functioning of others. Stated differently, *holding* appeared to beget more *holding*. This was evidenced by Jordan’s (asymptomatic participant) explanation: “*Life has enabled me to move on; love*, *people*, *purpose*, *a job*, *a family*”.

A reverse process was evident among the symptomatic sample. Commonly, symptomatic participants were unable to replicate the *holding* environment of the military. The experience of *rupture* was thus multiplicative. As an example, Tom (symptomatic participant) demonstrates this by describing the successive impacts of *rupture* from his deployment injury upon his family life, his ability to work and self-perception:

*“Yes, because I felt like a failure, I couldn’t support my family. Sometimes I still feel like that now and we struggle because obviously I’m classed as disabled as well now”.*

The symptoms of PTSD themselves were an additional engine of ongoing *rupture* that impacted the symptomatic, but not the asymptomatic, sample. Flashbacks and re-experiencing jolted traumatic memory into the present, whilst anger and personality changes negatively impacted the nurturing of balanced, positive, nurturing family relationships and avoidance led to a shrunken social circle. Dan (symptomatic participant) described: “*My friends*, *the circle just got smaller and smaller*. *So my standard of living was shockingly bad”*. In these cases, the family became a singular *holding* structure for the individual. In addition, by virtue of having to manage symptoms, symptomatic participants experienced unique *ruptures* such as the practical and emotional burdens of navigating formal support, encountering stigma, delays to diagnoses and treatment, disjointed care or a lack of adequate treatment.

It is worth noting that cumulative *rupture* was not reported by all symptomatic participants. Freddie (symptomatic participant), for instance, was managing symptoms whilst working for a blue-light service and reported, *“I always had that friendship network and that family network outside that I could draw on”*.

## Discussion

The current study explored how post-traumatic stress may develop among a group of former UKAF Army and Royal Marine personnel who previously deployed in combat roles to Iraq and Afghanistan. Our findings suggested that the military functioned as a temporary container for *holding ruptures* experienced on deployment(s). When participants left the military, they lost access to various practical, cultural, social and ethical scaffolds; most notably, the military collective and contexts where traumatic experiences made sense. For symptomatic participants, leaving the military elicited a revisitation, or exacerbation, of post-traumatic stress, and was the juncture at which participants consciously joined up their distress and experiences on deployments. Participants that were asymptomatic were more able to preserve and recruit new *holding* supports once leaving the military. This was further made possible by the absence of deleterious symptoms that may *rupture* said supports, e.g. flashbacks, avoidance and emotional withdrawal. Overall, the concepts of *holding* and *rupture* could offer an explanatory framework for the worsening outcomes of ex-military personnel and has potential relevance for explaining the onset of a range of other psychological problems, although further research is required to determine its value in relation to other groups and contexts.

A main finding of the present study was the worsening, or re-emergence, of deployment-related post-traumatic stress in the years after military-to-civilian transition. The following discussion will excavate three potential reasons for this. The first relates to the military’s *holding* capacities, which echoes the psychoanalytic processes described by Winnicott’s ‘holding’ [52] and Bion’s ‘containment’ [53]. Both represent different aspects of the insulating environment caregivers generate to help contain, transform and co-process an infant’s distress. These functions may be imitated in part by the pseudo-familial structures of the military, such as its paternalistic leadership and social networks. Indeed, comradeship has been consistently identified as supportive of wellbeing and protecting against the development of mental health problems [54, 55]. Whilst social support can help individuals to feel understood, to process and make meaning of traumatic experiences [29], our findings indicate that the military collective might also act as a shock-absorber which cushions and, in some cases, postpones individual impacts from being realised until years after the traumatic exposure. This might offer some interpretation to the results of an Israel veteran study which showed how higher levels of social resources were associated with longer delays in the onset of PTSD [56]. It is possible then that the individuating/ individualising process of leaving the military allows for deployment experiences to be registered on a personal level.

A second explanation of the re-emergence of post-traumatic stress refers to the stress of the military-to-civilian transition. This can include finding and adjusting to civilian employment, re-establishing a social circle, changes to family life, potential psychological difficulties and/or physical injuries [54, 57, 58], and feelings of grief, loss and identity-change [5, 59]. Indeed, the course of PTSD symptomatology fluctuates, with particular flares at times of adversity [60, 61]. This kind of triggering or reactivation is noted in other research [62–64]. In addition, it is possible that some psychological presentations may not be as noticeable, or even impairing, in certain contexts. Stated differently, some symptoms may be disruptive to functioning in some environments but manageable, or indeed productive, in others. For example, low levels of emotional numbing and hypervigilance may indeed be productive on deployment, e.g. *“the hyperarousal that was often key to life-saving instantaneous recognition of hidden improvised explosive devices and ambushes ‘downrange’ is now maladaptive and psychopathologic”* (p. 701) [65]. However, the possibility that these kinds of psychological defences may facilitate further exposure to traumatic experience (i.e. continuing to deploy) is potentially problematic since cumulative exposure to traumatic events is a well-established risk factor of PTSD [66].

Thirdly, the intensification of post-traumatic stress after military-to-civilian transition could be explained by profound shifts in one’s cultural and ethical ecology. Traumatic events compel individuals to reckon with the meaning of what has happened [22, 29] and such meanings are necessarily informed by the social worlds we inhabit: “*to regain their footing*, *people often turn to culturally available practices*, *symbols*, *and structures to help reorient them to the world*” (p. 753) [67]. In the current study, we highlighted how military training and culture provides a range of ethical, linguistic and cultural sense-making frameworks, from collective values [3, 45] to operational terminologies, which critical military work cites as a method to create, and dehumanise, the ‘enemy’ [68]. Whilst in the military, individuals are situated in specific cultural repertoires that give rise to, what Larner et al., (2011) calls, ‘constructive coherent meanings’ about the military’s interventions and praxis. Yet, these meanings may not easily transfer to, or survive in, other contexts [29], particularly if value and belief systems widely diverge. Bergman et al., (2014) [3] research term this a ‘reverse culture shock’, a disorientation that comes with moving back into a civilian context. Smith & True (2014) similarly highlighted the dissonance between the military’s requirement for deindividuation, hierarchy and dissociation and the civilian expectation to be autonomous and relational, which can amount to a ‘warring identity’ [69].

By adopting a phenomenological orientation, the present study could highlight a specific stage in the acknowledgement of post-traumatic stress, namely individuals’ processes of ‘conscious realisation’¸ namely nightmares, flashbacks, questioning of one’s experiences and re-experiencing of conflict. The intensity of these experiences once leaving the military could be interpreted as a call to reconsider the meaning of deployment experiences in light of this radical context change. Larrabee (1995) [39] notes:

*“The eruption of trauma memories as an insistent occurrence of PTSD symptomology might be the demand "by" that past experiencing for an open questioning that will strive for a new manner of interconnection. The original emergence of the PTSD "symptoms," is the beginning of such "questioning" on the part of one’s consciousness itself (remember to take consciousness here in a very broad sense)… The eruption of PTSD trauma memories signals the consciousness’ readiness for this working through, a readiness that might not seem obvious to the PTSD sufferer in the original phases of symptomology.”* (p.361)

The reignition of deployment trauma once leaving the military is perhaps not surprising given both beckon a fundamental reorientation of one’s sense of self and existence (what Scarry calls the ‘unmaking’ and ‘remaking’ of worlds) [27, 67].

Overall, the present study explored how post-traumatic stress develops from a lived perspective. The narratives of participants about their symptoms and experiences were retrospective. Since research interviews often call for participants to improvise a life-story, participants may have engaged in some ‘narrative smoothing’ [70] whereby life events are presented coherently and/or reframed based on what is known now in the present. Despite this, there were noticeable commonalities in how participants described post-traumatic stress developing over time.

Overall, post-traumatic stress seemed to emerge intermittently/ gradually and was managed for most of the time participants were in the military. A retrospective study of treatment-seeking UK ex-serving personnel similarly found that ‘full’ disorder manifested on average 14 months after leaving the military [71]. We presented the development of post-traumatic stress using the terms ‘compartmentalisation’, ‘episodic’ collapse’ and ‘conscious realisation’. These phases resonate strongly with the stages observed among Swiss civilian inpatients with complex PTSD (C-PTSD) [72]. Here, Stadtmann et al. (2018) outlined an initial stage of emotional ignorance, e.g. patients experienced some signs of post-traumatic stress, but did not connect them to trauma. This was followed by overcompensation, where patients attempted to subconsciously control their symptoms, primarily using dissociation and exhibited a high level of functioning at school and work. Lastly, paroxysm, which related to an outburst, or sudden increase, of symptoms marked an exhaustion of their compensatory strategies, which gave way to new perspectives.

Since the participants we interviewed recounted multiple traumatic experiences, it is possible that the stages of post-traumatic stress we identified are indicative of other PTSD typologies, e.g. c-PTSD or delayed-onset PTSD. We did not measure c-PTSD or delayed-onset PTSD in this sample, however these presentations could be particularly relevant for cohorts who may have developed long-term adaptations to traumatic exposure deployed throughout the peacekeeping and combat operations of the 1990s-2010s. Given the poorer treatment response in military groups compared to civilians [70, 71], further investigation into these typologies of PTSD, and the timing and sequence of symptoms, in those with multiple exposures would be worthwhile.

### Implications

Our analysis provides a framework for why the asymptomatic and symptomatic samples within the present study reported different outcomes in PTSD. The cumulative nature of *holding* and *rupture* in the current study is harmonious with the theory of cumulative advantage/ cumulative disadvantage [73]. Over time, the chain of positive outcomes experienced by the asymptomatic participants produced “*successive increments of advantage such that the gaps between the haves and the have-nots … widen*” [74] (p. 606). Asymptomatic participants further do not navigate the burden of experiencing and managing symptoms or of the need to seek help. *Holding* and *rupture* may thus offer valuable conceptual tools for describing longitudinal processes relating to health, wellbeing, identity and a range of other psychological processes, yet needs further exploration and development in other applications to determine its reach and fit as a more general framework.

Findings further highlighted how the development of PTSD relies upon the wider milieu, and interactions between, one’s employment, relationships, health, and quality of life. Interventions targeting the management of, or recovery from PTSD, must take into account the quality of individual’s social networks, relationships, their economic agency, community inclusion, sense of purpose and meaning, in addition to minimising any unnecessary ruptures caused by the delays and disruptions to mental health treatments. As found in other studies [36, 75, 76], participants’ spouses/partners performed an essential, and sometimes singular, *holding* function during deployment, transition and in the post-military years in helping participants manage their symptoms. This emphasises the need to factor in the family in the planning of supports both during and after personnel’s military employment.

Our findings further indicate that whilst deployment and military-to-civilian transition represent discrete and time-bound phases, impacts of these experiences may reverberate and interact with one another through the lifespan. Provisions supporting the military-to-civilian transition should incorporate focused care and attention upon the potential re-emergence of deployment-related stress for those with histories of deployment.

Overall, nuances in the present analysis showed that *holding* supports within the military may not always be conducive to long-term wellbeing. Examples included the military collective, alcohol use, and the thrill, distraction and purpose provided by deployments. All factors potentially inhibited the real-time processing of traumatic deployment events for some participants. The main example of this in the present study was psychological compartmentalisation. Compartmentalisation was variably regarded as a military skill and identified as one of the reasons for some asymptomatic participants’ ‘resilience’, as well as a precondition of post-traumatic stress. The compartmentalisation of stress is a natural, and necessary, defence and forms of emotional detachment are essential for trauma-exposed occupational groups [77]. From qualitative descriptions alone, it is unclear if there is a structural distinction between compartmentalising and dissociative processes, e.g between 1) symptomatic participants ‘boxing’ off and b) asymptomatic participants ‘shelving’ their traumatic experiences. Further research into the nature of compartmentalisation, and its long-term effects, in larger samples would shed light on the role this may play in the inception, or maintenance, of both PTSD and c-PTSD, particularly considering that peri-dissociation is an influential risk factor in the development of the disorder [78, 79].

The present study additionally found evidence that childhood adaptations to adversity, such as sensation-seeking, hypervigilance, emotional desensitisation, and tolerance to violence [80] might initially be useful in a military career, particularly on deployment. Those experiencing childhood disadvantage might be particularly attracted to a career in the military for educational opportunities and geographical, economic and social mobility [81], however the negative psychological impacts of experiencing multiple traumatic exposures throughout the lifespan is an essential issue that must inform the UK military’s stance on safeguarding and welfare. There is limited research on the compounding effects of childhood and military-related traumatic experiences among UK personnel and, taking into consideration the through-life interactions of life events evident in the present study, this area warrants more detailed investigation.

### Strengths and limitations

In the present study, ‘PTSD’ was inextricable from the rest of individuals’ life-contexts. Whilst the positivist, quantitative tradition is motivated to isolate variables during analysis, the entwinement of PTSD and its ethical, cultural contexts formed a particular strength of this analysis. Tracing the interplay of *holding* and *rupture* across many aspects of participants’ lives allowed us to capture the wider biographical and ecological quality of PTSD symptomatology. Doing so helped us to examine how so-called ‘big *T’* traumatic events (criterion A events such as combat experiences) are in contact and dialogue with ‘small *t’* events (more quotidian stressors). In this regard, the model of *holding* and *rupture* can itself perform as a ‘container’ that brings together biological, cognitive psychoanalytic, attachment-based and social and cultural theories about PTSD and even other mental health problems and psychological processes, such as grief, loss and identity changes [3, 22, 52, 53, 82]

The findings of the current study were developed from the interviews of 17 participants and may be specific to those with these military characteristics. Overall, we expect the findings could have some transferability to other UK and international military populations, however the findings presented refer to the particular experiences of the individuals interviewed and therefore further research is required to determine whether *holding* and *rupture* as presented in this article have relevance and resonance with other groups. One fundamental issue for transferability is, however, the absence of officers from the symptomatic group. This was the result of the premature termination of data collection due to the COVID-19 pandemic. As such, we have not been able to include experiences of symptomatic officers who may have higher socioeconomic status, and different experiences of military hierarchy, culture, and leadership responsibilities. The study does not explore how the processes described may vary by rank or levels of responsibility in depth, yet there was some evidence that specialism (e.g. attending to medical emergencies) and responsibility for the lives of others had an impact upon individuals’ meaning-making processes. These nuances would be valuable to research further in future work.

A fundamental challenge presented by the current study was the analytical conundrum of comparing the presence of post-traumatic stress against its absence. Were asymptomatic participants unaffected by their deployment experiences because they were able to maintain cognitive distance, were able to talk through these events and recruited diverse supports *or* was this only possible because they simply were not traumatised in the first place? There was some evidence of a circular, reciprocal process: that the damage of an event is mediated by buffers which themselves are impacted by the potential damage of an event. We have expressed these dynamics as an ‘interplay’ in the present study, however the ‘chicken or the egg’ temporal complexities inherent to PTSD would be worthy of further reflection and study.

## Conclusion

A qualitative exploration of post-traumatic stress among ex-military personnel of the UK Armed Forces showed that traumatic impacts from childhood and deployment may be temporarily buffered by the wrap-around supports of the military. The military-to-civilian transition, and the loss of military scaffolds may lead to a (re-)emergence of post-traumatic stress and deployment memories. It is crucial that the UK Ministry of Defence, service providers and military charities are cognizant of the potential intensification of traumatic impacts in the years following discharge for cohorts with long-term traumatic exposures and that symptoms may recur along the lifespan. Findings may have broader transferability to other psychiatric or psychological responses beyond PTSD, including, c-PTSD, other mental health problems, and grief, loss and identity change when leaving the military.

## Supporting information

S1 File Reflexivity statement. (DOCX)

S2 File Table of participants’ characteristics. (DOCX)

S3 File Interview guide. (DOCX)
