# Supplementary material for: Holding and rupture: Describing post-traumatic stress among former UK Army and Royal Marine personnel deployed to Iraq and Afghanistan
Source: PLoS One. 2024 Aug 9;19(8):e0308101. doi: 10.1371/journal.pone.0308101 (PMC11315309; doi:10.1371/journal.pone.0308101)
Supplement: S1 File — (DOCX) [file pone.0308101.s001.docx]

# Supporting information

# S1 Reflexivity statement

Findings from this research have been informed by the authors of the study, other members of the King’s Centre for Military Health Research (KCMHR). Implications and recommendations of the research were discussed in consultation with a Participant and Patient Involvement (PPI) group consisting of ex-serving personnel, some of whom with lived experiences of PTSD, and a group of stakeholders, including Ministry of Defence (MoD), National Health Service (NHS) and Third Sector professionals. The team consisted of non-military researchers and two military-connected clinicians (a currently serving mental health nurse and a formerly serving psychiatrist), and expertise were drawn from a forensic psychiatrist. Throughout the research process, efforts were made to reflexively interrogate the influence of the researchers’ positionality and experiences upon data collection, analysis and the interpretation of results (1,2).

As the principal analyst (LP), I reflected upon the split of having one foot in the ‘method’ and the other in the ‘life-world’ of the participant. Throughout data collection, I maintained a pragmatic posture to the interview process pragmatically and it was only when I was analysing the data that I entered a deeper engagement with the emotional qualities of the data. During process, I encountered a ‘stickiness’ to the participants’ narratives, which meant I regularly thought about them and imagined the deployment events they described. I noted there was some parallel process where my own relaitonship to the work was tracking a similar process of compartmentalision followed by conscious realisation (as reported in the study’s findings).

Due to the many “subterrean aspects of the interview relationship” (p. 21) (3), it is possible that unresolved, minimised, displaced and rejected sensations took root in the vessel of the interview(er). Indeed, in military qualitative research, the interview is often the first site participants get to ‘story’ their traumatic experiences. My experience in this work has given me a felt sense that qualitative researchers are not just instruments of the research process (4), but organs that might *metabolise* data during its iterative transformations. Co-authors and clinicians within KCMHR were supportive during this process and I acknowledge in particular the clinical supervision of Dr Deirdre MacManus.

**References**

1. Lazard L, McAvoy J. Doing reflexivity in psychological research: What’s the point? What’s the practice? Qualitative Research in Psychology. 2020;17(2):159-77.
2. Shaw R. Embedding reflexivity within experiential qualitative psychology. Qualitative research in psychology. 2010;7(3):233-43.
3. Roper M. Analysing the analysed: transference and counter-transference in the oral history encounter. The Journal of the Oral History Society. 2003;31(2):20-32.
4. Meloy JM. Writing the qualitative dissertation: Understanding by doing. Psychology Press; 2001.
