# Supplementary material for: Holding and rupture: Describing post-traumatic stress among former UK Army and Royal Marine personnel deployed to Iraq and Afghanistan
Source: PLoS One. 2024 Aug 9;19(8):e0308101. doi: 10.1371/journal.pone.0308101 (PMC11315309; doi:10.1371/journal.pone.0308101)
Supplement: S2 File — (DOCX) [file pone.0308101.s002.docx]

# Supporting information

# S2 Table of participants’ characteristics

| **Pseudonym** | **Rank** | **Branch of service** | **Age group (years) at interview** | **Age group (years) when enlisted** | **Length of military service in years** | **Areas deployed*** | **Military discharge** |
| --- | --- | --- | --- | --- | --- | --- | --- |
| Dan | NCO¥ | Army | 40-44 | 25-29 | 10 | Afghanistan | Medical - physical and mental health |
| Ali | Enlisted | Army | 30-34 | Under 18 | 5 | Iraq | PVR ± |
| Freddie | Enlisted | Army | 30-34 | Under 18 | 7 | Bosnia, Iraq and Afghanistan | PVR |
| Jimmy | Enlisted | Army | 30-34 | 19-24 | 4 | Afghanistan | PVR |
| Mike | NCO | Army | 45-49 | Under 18 | 23 | Bosnia, Iraq, Afghanistan | End of term |
| Tom | NCO | Army | 35-39 | Under 18 | 10 | Northern Ireland, Iraq and Afghanistan | PVR |
| Brandon | Enlisted | Army | 45-49 | Under 18 | 26 | Northern Ireland, Bosnia, Iraq and Afghanistan | Medical discharge - physical injury |
| Beth | NCO | Army | 40-44 | 19-24 | 16 | Bosnia, Iraq and Northern Ireland | Medical discharge - physical injury |
| Chris | NCO | Army | 50-54 | Under 18 | 23 | Northern Ireland, Bosnia and Iraq | End of term |
| Matthew | NCO | Army | 44-49 | Under 18 | 26 | Northern Ireland, First Gulf War, Bosnia, Iraq and Afghanistan | Medical discharge - physical injury |
| Keith | NCO | Royal Marines | 44-49 | Under 18 | 24 | First Gulf War, Northern Ireland, Iraq, Afghanistan | End of term |
| Tony | Enlisted | Army | 30-34 | Under 18 | 5 | Iraq | PVR |
| Craig | Enlisted | Royal Marines | 40-44 | 19-24 | 16 | Kosovo, Afghanistan | Medical discharge - physical injury |
| Rachel | Commissioned officer | Army | 35-39 | 20-24 | 6 | Iraq and Afghanistan | PVR |
| Jordan | Commissioned officer | Army | 35-39 | Under 18 | 10 | Kosovo, Northern Ireland, Bosnia, Afghanistan, Iraq | PVR |
| Steve | Enlisted | Army | 45-49 | Under 18 | 23 | Kosovo, Iraq, Afghanistan | PVR |
| Gareth | Commissioned officer | Army | 30-34 | Under 18 | 5 | Afghanistan | PVR |

**Details of operation names omitted for anonymisation, and deployment areas reflect those discussed in the interview*

± Premature Voluntary Release (PVR)

¥ Non-Commissioned officer (NCO)
