# Supplementary material for: Holding and rupture: Describing post-traumatic stress among former UK Army and Royal Marine personnel deployed to Iraq and Afghanistan
Source: PLoS One. 2024 Aug 9;19(8):e0308101. doi: 10.1371/journal.pone.0308101 (PMC11315309; doi:10.1371/journal.pone.0308101)
Supplement: S3 File — (DOCX) [file pone.0308101.s003.docx]

# Supporting information

# S3 Interview Schedule

**Note: This template provides a general structure of the interview. The questions were tailored based on the chronology of events described by participants during their background section.*

*The interviewer also used prompts regarding feelings, potential symptoms if described and followed up on details where necessary. The interviewer referenced feelings/ symptoms as worded by participants.*

| **Preparation and notifications** |
| --- |
| **Section 1 – Background** |
| *[Section used to develop timeline of reference points that can be referred to during the interview]*   - Can you tell me more about your service and role in the military over the years? - Which deployments were you involved in? - When did you join the military? - How would you describe your initial experiences upon joining? - How would you describe your experiences in your first unit? |
| **Section 2 – Military experiences** |
| **DEPLOYMENT STRESSORS:**   - Can you describe your role on deployment X? - How would you describe this deployment? - Can you describe any circumstances where you felt under threat? - Would you be able to describe when you witnessed harm to others? - Can you describe any other events that had an impact upon you? - How did you feel about this at the time? - Did you receive support from the military during your deployment? - Were you injured at any point of your deployment? - Were you aware of any difficulties for family, friends or others at home?   **POST-DEPLOYMENT**   - Can you describe your mindset when you returned from deployment? - What support did you receive from the military when you returned home? - What happened to your feeling of *X* in the months after your deployment? - Can you describe whether your time in X has had any impact on you in the present? - Can you describe if there are any times you feel more affected by what you experienced? - *[If relevant]* Did you seek help and what was this like?   **NON-DEPLOYMENT STRESSORS**   - Were there any other events happening at the time that caused stress? - Can you describe what this was like and how this felt at that time? - How would others describe your behaviour at this time? - What impacts did this have upon other areas of your life?   **WHEN SYMPTOMS DESCRIBED**   - If symptoms mentioned: how did you understand these experiences? - When did these arise? [*In reference to events]* and how long did they last for? - Can you describe if you sought any help for this at the time (*prompts: why/ why not?)*   *[For time in the military: Repeat non-deployment and deployment sections over the biography]* |
| **Section 3 – Post-military experiences** |
| - Can you describe more about the circumstances of you leaving service? - What was your experience approaching discharge? - How would you describe your transition overall? - Can you describe how you felt at the time? - What happened in the years after you resettled? - Can you describe what it was like for you during these years? - Did you access any support during this time? - Can you describe your current living situation? - How would you describe how you’re feeling now? - [*If relevant]* You mentioned [feelings/ symptoms], how would you describe these now and their impact? |
| **Section 4 – Childhood factors** |
| - What was it like to live in the community you grew up in? - Who was at home while you were growing up? - Were there any specific difficulties you remember about growing up? - Can you describe if there were any other events in your childhood that have caused you problems later on in life? - Are there any other factors in your background that you think might be linked to (symptoms/feelings)? - What other factors might be linked to your (symptoms/feelings)? |
| **Section 5 - Final reflections** |
| - How do you consider your military service to have affected you in the present? [*prompts: can you describe the positive/ negative consequences of having served?]* - Are there any additional comments or observations to make which we haven’t covered? |
| **Closing** |
